# Explainable machine learning model for predicting spontaneous bacterial peritonitis in cirrhotic patients with ascites

**DOI:** 10.1038/s41598-021-00218-5

**Published:** 2021-11-04

**Authors:** Yingying Hu, Ruijia Chen, Haibing Gao, Haitao Lin, Jinye Wang, Xiaowei Wang, Jingfeng Liu, Yongyi Zeng

**Affiliations:** 1 Department of Pharmacy, Mengchao Hepatobiliary Hospital of Fujian Medical University, Fuzhou, 350025 China; 2 Department of Infection and Liver Diseases, Mengchao Hepatobiliary Hospital of Fujian Medical University, Fuzhou, 350025 China; 3 The Big Data Institute of Southeast Hepatobiliary Health Information, Mengchao Hepatobiliary Hospital of Fujian Medical University, Fuzhou, 350025 China; 4 Department of Hepatopancreatobiliary Surgery, Mengchao Hepatobiliary Hospital of Fujian Medical University, Fuzhou, 350025 China

**Keywords:** Liver cirrhosis, Machine learning, Computational models

## Abstract

Spontaneous bacterial peritonitis (SBP) is a life-threatening complication in patients with cirrhosis. We aimed to develop an explainable machine learning model to achieve the early prediction and outcome interpretation of SBP. We used CatBoost algorithm to construct MODEL-1 with 46 variables. After dimensionality reduction, we constructed MODEL-2. We calculated and compared the sensitivity and negative predictive value (NPV) of MODEL-1 and MODEL-2. Finally, we used the SHAP (SHapley Additive exPlanations) method to provide insights into the model’s outcome or prediction. MODEL-2 (AUROC: 0.822; 95% confidence interval [CI] 0.783–0.856), liked MODEL-1 (AUROC: 0.822; 95% CI 0.784–0.856), could well predict the risk of SBP in cirrhotic ascites patients. The 6 most influential predictive variables were total protein, C-reactive protein, prothrombin activity, cholinesterase, lymphocyte ratio and apolipoprotein A1. For binary classifier, the sensitivity and NPV of MODEL-1 were 0.894 and 0.885, respectively, while for MODEL-2 they were 0.927 and 0.904, respectively. We applied CatBoost algorithm to establish a practical and explainable prediction model for risk of SBP in cirrhotic patients with ascites. We also identified 6 important variables closely related to the occurrence of SBP.

## Introduction

Spontaneous bacterial peritonitis (SBP) is the most common life-threatening infection in patients with ascites due to liver cirrhosis. Early and accurate diagnosis is key to improving patient survival. The early clinical symptoms of most patients with SBP are not typical, so it is easy to be misdiagnosed, which has an effect on the early treatment of patients^1^. It is very important to actively look for evidence of SBP diagnosis. Although diagnostic abdominal puncture can provide evidence for early diagnosis of SBP, it is an invasive procedure. With increasing use of antibiotics, there is a gradual shift in the causative flora of SBP from Gram-negative bacteria to Gram-positive and, more importantly, to drug-resistant bacteria. It is necessary to reduce the misdiagnosis rate of SBP, rational use of antibiotics, in order to prevent the development of drug-resistant bacteria. As such, there is a real need to develop a sensitive, specific, and easy-to-apply non-invasive methods to diagnose SBP or to exclude SBP.

Systemic inflammatory response for SBP is complex, and a single indicator is insufficient for diagnosis; a prediction model derived from a combination of different parameters would be more accurate. In recent years, people had been trying to explore the prediction model or scoring system for predicting risk for SBP in patients with cirrhotic ascites^2–8^, but so far there was no recognized standard model. Machine learning (ML) is a discipline that uses computational modeling to learn from data, meaning that performance at executing a specific task improves with experience (i.e., more data). Machine learning has been applied in many fields of medicine such as outcome prediction, diagnosis, medical image interpretation, and treatment^9^. Electronic health records are increasingly becoming not only repositories of healthcare data, but platforms that can be used to deploy ML models as tools to help guide clinical decision-making. Although complex machine learning models are commonly outperforming the traditional simple interpretable models, it is often criticised for being a black-box model, which makes clinicians find it hard to understand and trust these complex models. Interpretability of models are crucial in medical environments where results have to be explained to medical providers to be widely accepted^10,11^.

In this study, we applied machine learning methods to predict the risk for SBP in patients with cirrhotic ascites, so as to realize the early diagnosis of first episode of SBP. After a comprehensive evaluation of catboost importance matrix plot, SHAP summary plot and doctors' clinical experience, we chose the influential variables and create the high performing models of dimension reduction, which not only saves computing time, but also is more suitable for clinical application. In addition, we used explainable machine learning methods to provide interpretability of the black-box for clinicians and patients^10,11^.

## Results

### Characteristics of patients

A total of 1399 inpatients who met the enrollment criteria were included in the model construction, of which 538 (38.5%) patients were identified as having cirrhosis ascites complicated with SBP. All patients were randomly divided into training set (n = 951) and validation set (n = 448) in a 7:3 ratio. The prevalence of SBP was 37.7% in the training set and 40.0% in the validation set. In training set, Table 1 depicted the relationship between the 46 variables we included and the incidence of SBP. Patients with cirrhosis ascites combined with hepatic encephalopathy and acute(sub-acute)-on-chronic liver failure were more likely to have SBP, but there was no significantly different in patients with esophagogastric variceal bleeding. A total of 26 laboratory variables were found to be statistically significant.

**Table 1:** Demographic and clinical features of the training set.

Variables	Spontaneous bacterial peritonitis		*P* value
	No (n = 592)	Yes (n = 359)	
**Gender**			0.349
Male	419 (70.8%)	265 (73.8%)	
Female	173 (29.2%)	94 (26.2%)	
Age, years	52.4 ± 12.1	52.6 ± 12.7	0.749
**Etiology of liver disease**			0.106
HBV-related cirrhosis	368 (62.2%)	209 (58.2%)	
Alcoholic cirrhosis	75 (12.7%)	47 (13.1%)	
HBV and Alcohol—related cirrhosis	45 (7.6%)	45 (12.5%)	
Cryptogenic cirrhosis	39 (6.6%)	29 (8.1%)	
Autoimmune cirrhosis	36 (6.1%)	12 (3.3%)	
Other	29 (4.9%)	17 (4.8%)	
**Esophagogastric variceal bleeding**			0.768
No	539 (91.0%)	324 (90.3%)	
Yes	53 (9.0%)	35 (9.7%)	
**Hepatic encephalopathy**			< 0.001
No	565 (95.4%)	319 (88.9%)	
Yes	27 (4.6%)	40 (11.1%)	
**Diabetes**			0.836
No	538 (90.9%)	324 (90.3%)	
Yes	54 (9.1%)	35 (9.7%)	
**Hypertension**			0.426
No	546 (92.2%)	325 (90.5%)	
Yes	46 (7.8%)	34 (9.5%)	
**Smoking history**			0.32
No	471 (79.6%)	275 (76.6%)	
Yes	121 (20.4%)	84 (23.4%)	
**Drinking history**			0.119
No	452 (76.4%)	257 (71.6%)	
Yes	140 (23.6%)	102 (28.4%)	
**Family history of liver cancer**			0.51
No	406 (68.6%)	238 (66.3%)	
Yes	186 (31.4%)	121 (33.7%)	
**Acute(sub-acute)-on-chronic liver failure**			
No	571 (96.5%)	305 (85.0%)	< 0.001
Yes	21 (3.5%)	54 (15.0%)	
C-reactive protein (mg/L)	7.71 ± 13.7	18.4 ± 30.7	< 0.001
Procalcitonin (ng/ml)	0.425 ± 2.55	0.612 ± 2.60	0.279
White blood cell count (× 10^9^/L)	4.48 ± 2.26	5.70 ± 3.71	< 0.001
Red blood cell count (× 10^12^/L)	3.84 ± 0.784	3.64 ± 0.825	< 0.001
Mean corpuscular volume (fL)	93.4 ± 10.0	93.7 ± 10.2	0.702
Hematocrit(%)	34.6 ± 9.77	31.7 ± 10.7	< 0.001
Neutrophil ratio (%)	60.2 ± 12.2	66.3 ± 12.9	< 0.001
Lymphocyte ratio (%)	29.4 ± 11.0	23.0 ± 11.4	< 0.001
Monocyte ratio (%)	7.53 ± 2.59	8.10 ± 3.04	0.00357
Hemoglobin (g/L)	119 ± 26.0	113 ± 26.2	0.00219
Mean hemoglobin (pg)	31.0 ± 3.98	31.3 ± 3.98	0.186
Mean hemoglobin concentration (g/L)	331 ± 17.4	334 ± 18.3	0.0102
Platelet (× 10^9^/L)	95.7 ± 56.2	98.5 ± 70.2	0.51
Mean platelet volume (fL)	10.6 ± 1.33	10.5 ± 1.48	0.095
Total protein (g/L)	64.2 ± 14.1	48.0 ± 24.3	< 0.001
Alanine aminotransferase (U/L)	159 ± 362	222 ± 494	0.0356
Aspartate aminotransferase (U/L)	158 ± 314	216 ± 394	0.017
Gamma glutamyl transpeptidase (U/L)	169 ± 245	181 ± 298	0.526
Alkaline phosphatase (U/L)	142 ± 85.7	154 ± 105	0.0814
Total bilirubin (µmol/L)	66.4 ± 88.0	134 ± 159	< 0.001
Direct bilirubin (µmol/L)	37.4 ± 57.3	77.5 ± 94.9	< 0.001
Total bile acid (µmol/L)	80.3 ± 89.5	127 ± 126	< 0.001
Cholinesterase (U/L)	3860 ± 1690	2870 ± 1290	< 0.001
Lactate dehydrogenase (U/L)	252 ± 207	278 ± 170	0.0415
Creatine kinase (U/L)	134 ± 163	157 ± 190	0.0548
Total cholesterol (mmol/L)	3.94 ± 1.48	3.39 ± 1.60	< 0.001
Triglyceride (mmol/L)	1.16 ± 0.742	1.18 ± 1.03	0.74
High-density lipoprotein(mmol/L)	0.965 (0.518)	0.674 (0.498)	< 0.001
Apolipoprotein A1 (g/L)	1.07 ± 0.403	0.792 ± 0.404	< 0.001
Apolipoprotein B (g/L)	0.793 ± 0.330	0.747 ± 0.316	0.0339
Urea (mmol/L)	4.90 ± 3.22	5.29 ± 4.13	0.127
Serum creatinine (µmol/L)	76.1 ± 66.2	79.2 ± 67.7	0.498
Uric acid (µmol/L)	307 ± 116	289 ± 135	0.0332
Prothrombin activity (%)	69.6 ± 19.3	56.2 ± 19.7	< 0.001
Sodium (mmol/L)	139 ± 3.46	136 ± 5.04	< 0.001

Data are represented as n (%) and mean ± SD.

### Construction of models

**Figure 1:**
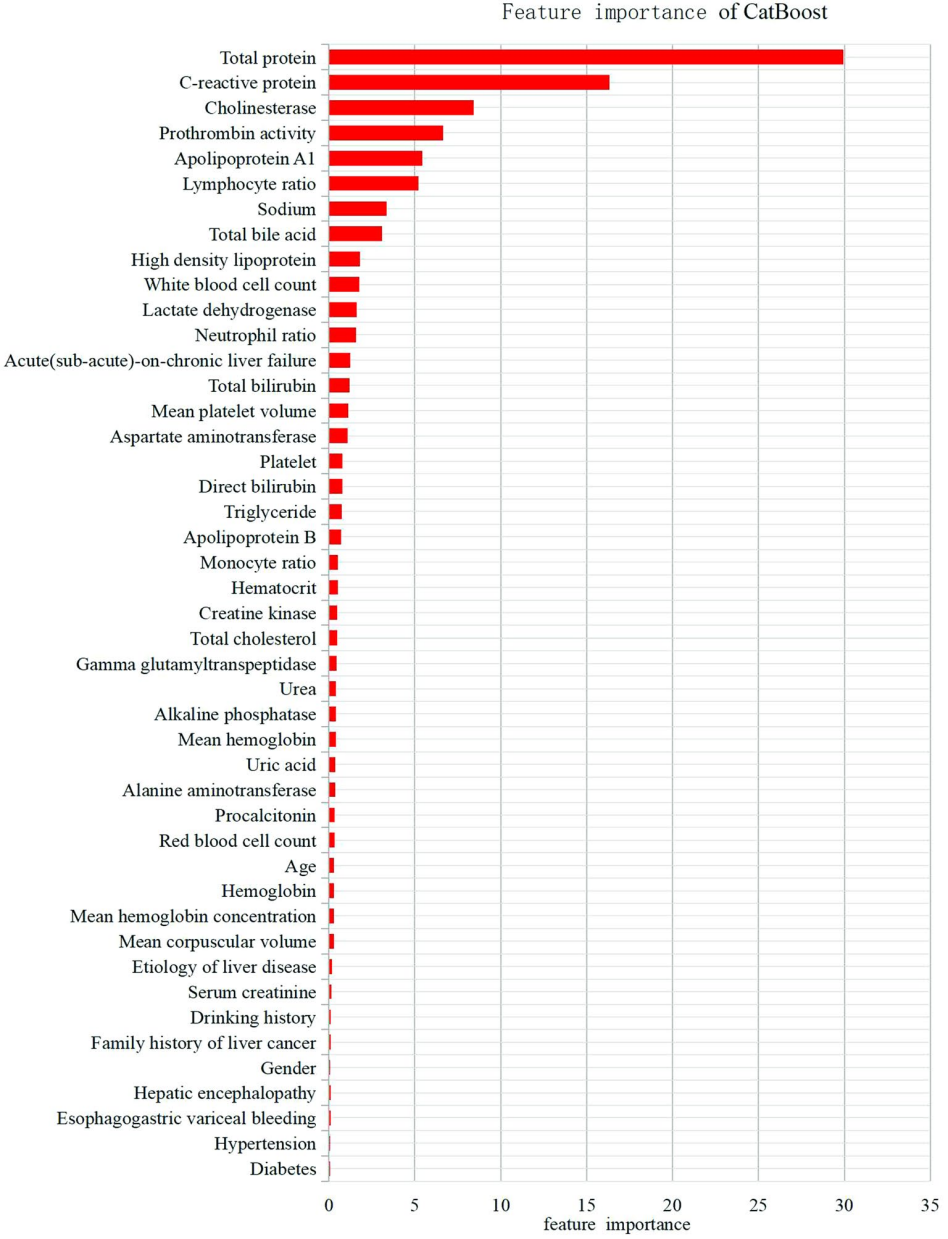
Importance matrix plot of the CatBoost model.

**Figure 2:**
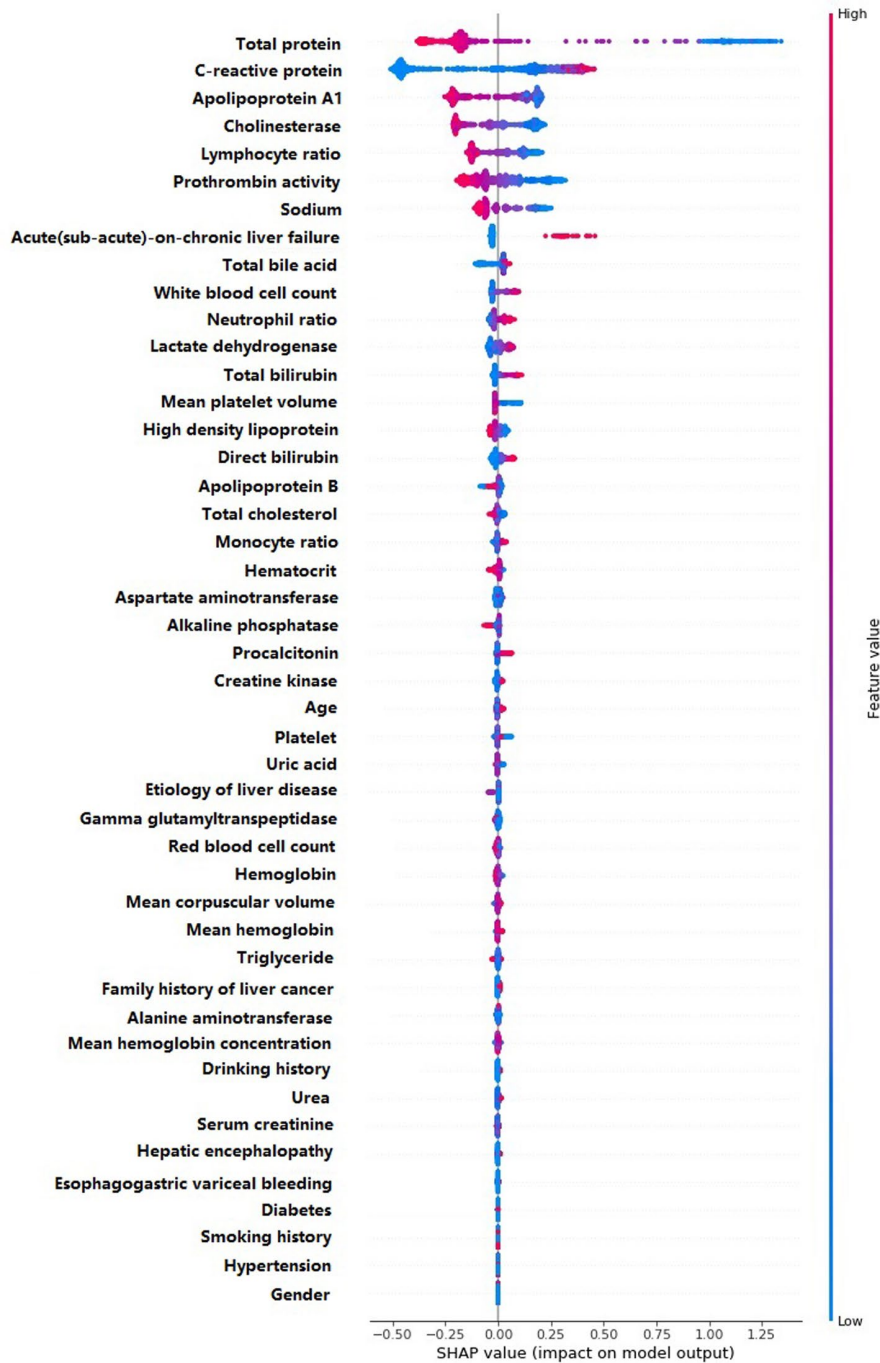
SHAP summary plot of the CatBoost model.

The MODEL-1 with 46 variables was constructed by CatBoost algorithm, and the AUROC in the validation set was 0.822 (95% confidence interval [CI] 0.784–0.856). Figure 1 showed the CatBoost's own feature importance matrix plot and Fig. 2 showed the SHAP summary plot for CatBoost. Through the feature importance ranking of the two graphs, we could well distinguish which were the importance variables affecting the model. The CatBoost tree visualization could also more intuitively let us understand the operation mechanism of the model (see Fig. S2 in Supplementary information). The SHAP summary plot showed the impact of each variable on the predicted outcome. For example, Figs. 1 and 2 both found total protein and C-reactive protein, to be the most important variables. In SHAP summary plot, higher total protein (red dots) is associated with lower incidence of SBP (SHAP value less than zero). Higher CRP (red dots) is associated with higher incidence of SBP (SHAP value greater than zero).

According to the CatBoost importance matrix plot and the SHAP summary plot, we obtained 11 variables that have the most influence on the model. Nine models were constructed through feature selection, and the AUROC values of each model were compared (see Supplementary information, Table S2). We found that the AUROC = 0.822 (95% CI 0.783–0.856) of the model with 6 variables was equivalent to MODEL-1, and it was the simplest and optimal model. After communicating with clinical experts, it was considered that these 6 variables not only had clinical diagnostic significance, but also could be easily collected in the clinical settings and reduced the missingness of values. Therefore, we constructed MODEL-2 using these 6 predictive variables: total protein, C-reactive protein, prothrombin activity, cholinesterase, lymphocyte ratio and apolipoprotein A1.

### Comparison of models performance

**Figure 3:**
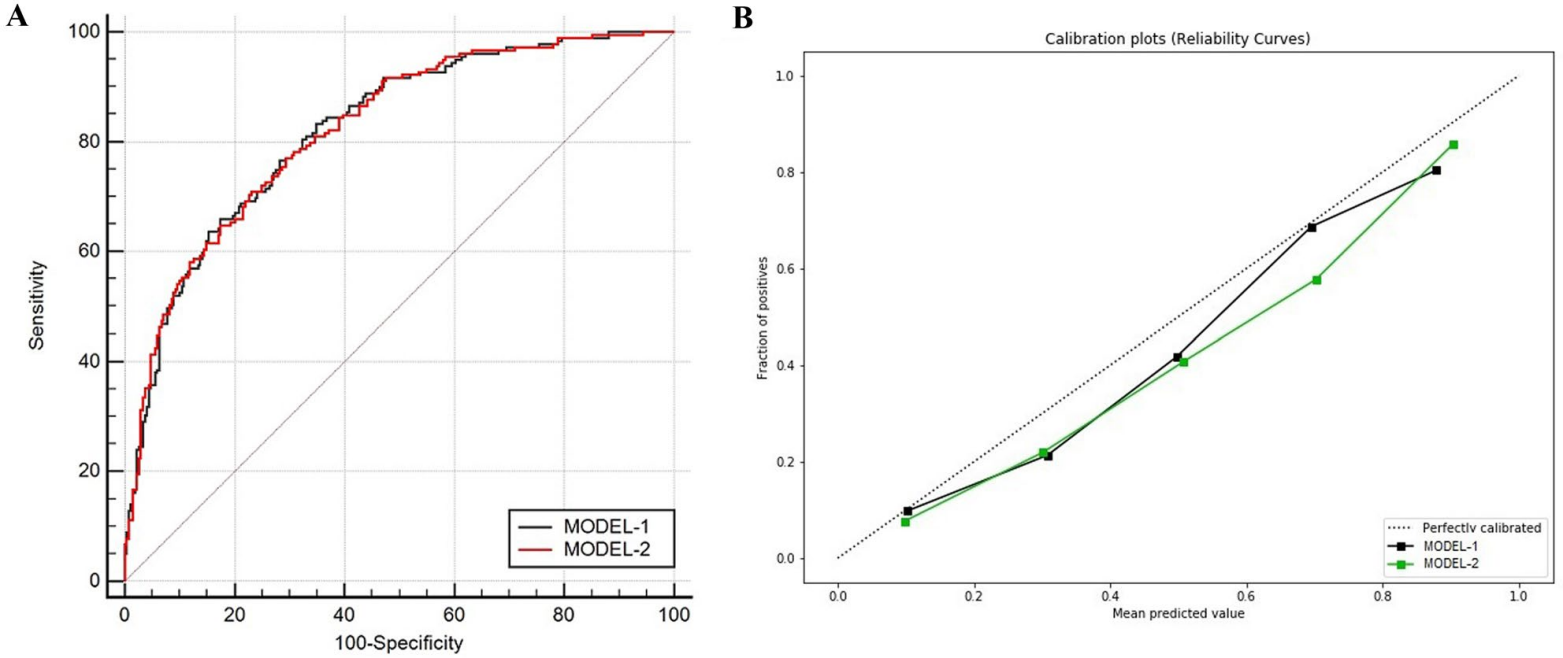
Receiver operator characteristic (ROC) curves and calibration curves for MODEL-1 and MODEL-2(validation set). (**A**) ROC curves showing the prediction performance of the MODEL-1 and MODEL-2. (**B**) Calibration curve reflecting the degree of consistency between the predicted risk and the actual risk of the MODEL-1 and MODEL-2.

The receiver operating characteristic curves in Fig. 3A showed that the AUROC of MODEL-2 was similar to that of MODEL-1. The calibration curve in Fig. 3B showed that the predicted values of our two models were close to the actual observation results. The performance of MODEL-2 was no worse than that of MODEL-1.

Table 2 showed performance of MODEL-1 and MODEL-2 as binary classifiers. By choosing the threshold probability that maximizes the F2-score of each model, the sensitivity of MODEL-1 and MODEL-2 were all greater than 0.89, and the negative predictive values were 0.885 and 0.904, respectively. Therefore, patients classified as low-risk by these models were less likely to develop SBP.

**Table 2:** Summary of prediction results of models on the validation set.

Value	Models	
	MODEL-1	MODEL-2
AUC	0.822	0.822
95% CI of the AUC	0.784–0.856	0.783–0.856
F2-score	0.840	0.815
Threshold	0.376	0.306
Sensitivity	0.894	0.927
Specificity	0.543	0.457
Positive predictive value	0.565	0.532
Negative predictive value	0.885	0.904

### Model explainability results for four patients

**Figure 4:**
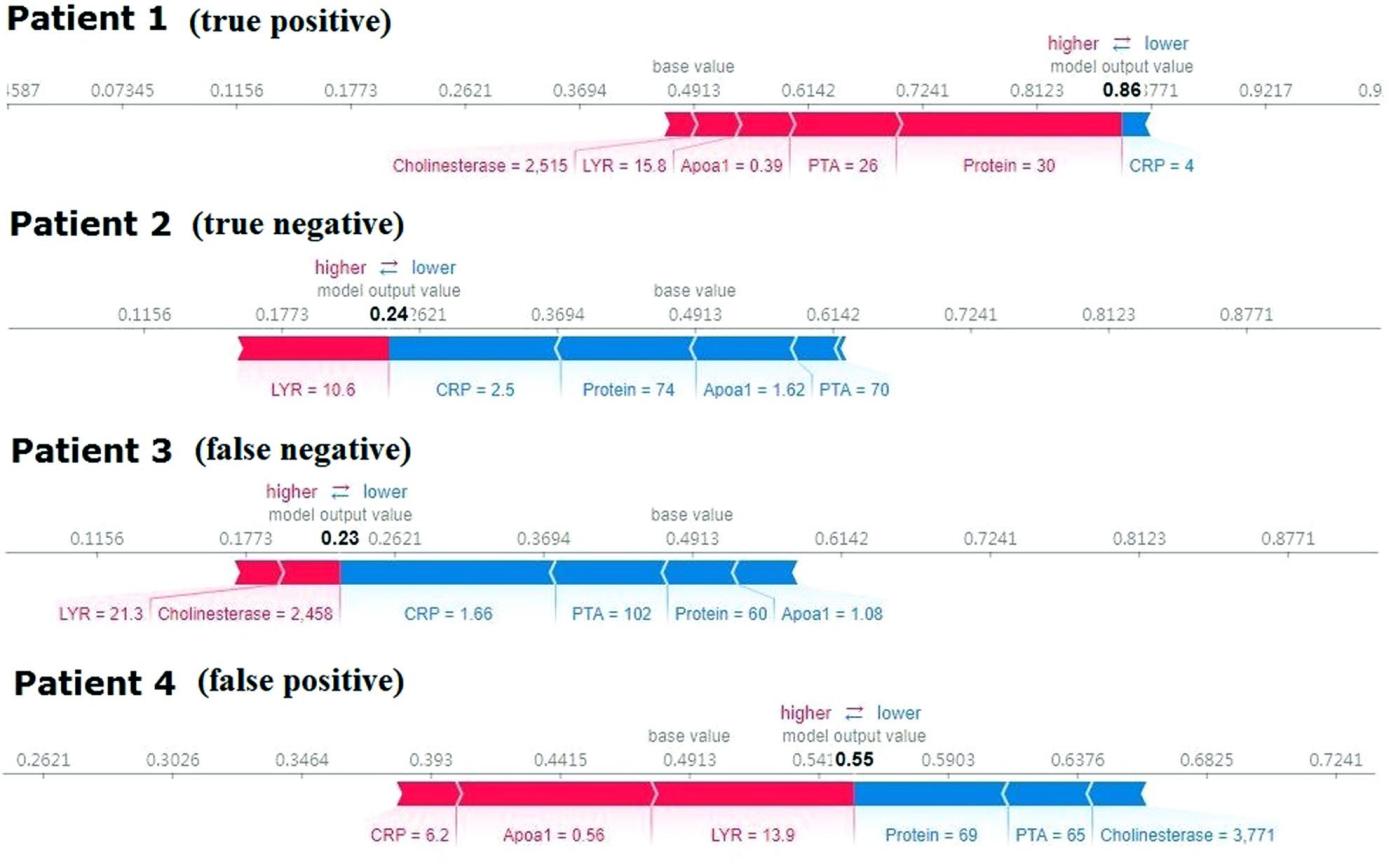
SHAP explanation force plot for 4 patients from the validation set of the CatBoost model. Protein, total protein; CRP, C-reactive protein; PTA, prothrombin activity; LYR, lymphocyte ratio; Apoa1, apolipoprotein A1.

Through the SHAP force plot in Fig. 4, some examples were given to illustrate the role of the SHAP method in explaining the machine learning model. The base value in the figure was equal to 0.4913, which meant that through MODEL-2, we predicted that the incidence of SBP in the validation set was 49.13%. And its SBP incidence was actually 40%, which meant that our model overestimated the risk. This phenomenon can also be observed in the calibration curve of Fig. 3B, that was, the fitting curve of the models were below the reference curve.

With the help of Fig. 4, we could intuitively judge that patient 1 was a cirrhotic ascites patient with SBP, and the actual result was the same (true positive). The model predicted that no SBP occurred in the patient 2, and the actual results was also no SBP (true negative). For patient 3, the model incorrectly predicted outcome as no SBP for the patient, whereas the actual outcome was SBP (false negative). Patient 4 was also incorrectly judged to have SBP (false positive).

### Model generalization ability evaluation

Among 1011 patients with cirrhotic ascites complicated with infection other than SBP, 578 (57.17%) were patients with SBP complicated with other infection, and the remaining 433 (42.83%) were patients with other infection alone. The AUROC of MODEL-1 was 0.809 (95% CI 0.783–0.833) and that of MODEL-2 was 0.803 (95% CI 0.777–0.827). It could be seen that both MODEL-1 and MODEL-2 have good generalization ability. The models we constructed excluding the factor of other infections could also well predict the occurrence of SBP in patients with cirrhotic ascites complicated with other infections. These fully reflected the clinical applicability of the models.

## Discussion

SBP was the main cause of death in patients with end-stage liver disease. In recent years, with the early diagnosis and effective use of antibiotics, the mortality related to SBP infection has decreased significantly. However, due to the extensive use of antibiotics, not only the bacterial spectrum of ascites infection changed, but also the production of multidrug-resistant strains increased, which seriously affected the effect of anti infection treatment and the prognosis of patients. In order to minimize the occurrence of bacterial resistance, it is wise to improve the diagnostic level of SBP and reduce the use of antibiotics for the low-risk population of SBP. In this single-center study, we used CatBoost algorithm to build a prediction model of SBP in cirrhotic ascites, solely based on the pre-paracentesis objective variables. Through the predictive model, we hoped to identify those patients with low risk of SBP, so as to reduce the use of antibiotics and invasive abdominal puncture. Therefore, the binary classifier generated by using the maximized F2-score of the model to set the threshold is more suitable for clinical application. In the clinical setting, recall is more important than precision. F2-score is conducive to improve the sensitivity of the model and reduce the false negative rate. Ikemura et al.^12^ better distinguished those COVID-19 infected patients at high risk of death by using F2-score, so as to give these patients more intervention and attention. Therefore, the binary classifier based on F2-score can improve the clinical application of machine learning model. In our validation set, the binary classifier based on F2-score made the negative predictive value of MODEL-2 0.904 and the sensitivity 0.927. This meant that our model can effectively identify low-risk patients with SBP. For these patients, we suggest that it is not necessary to carry out abdominal puncture and the use of antibiotics, so as to reduce the occurrence of adverse events caused by invasive operation and delay the occurrence of bacterial resistance.

The MODEL-1 constructed with 46 variables and the MODEL-2 constructed with 6 variables had good prediction performance. This showed that our dimensionality reduction was successful and 6 variables alone were still able to generate high performing models. Dimensionality reduction is an important process in machine learning model development. Data set dimensionality reduction can not only speed up the calculation, but also remove some redundant variables to solve the multicollinearity problem. The more important advantage of dimension reduction is to facilitate the application of clinicians in daily work and reduce the lack of data, thus reducing the risk of bias from imputation. The reduced dimension model is also helpful for other researchers to reproduce our work with their unique cohorts and realize the popularization and application of the model. In addition, through the evaluation of the generalization ability of the model, it was proved that our models can also well predict the occurrence of SBP in patients with cirrhotic ascites complicated with other infections. These fully reflected that the model had good robustness and clinical applicability, which was conducive to its popularization and application.

The 6 variables in MODEL-2 were important characteristics for predicting the first SBP in cirrhotic patients with ascites. Previous studies have confirmed that severe liver damage (Child–Pugh grades B/C) was a high risk factor for SBP^13,14^, and there was a correlation between the first SBP episode and liver dysfunction^2,15^. In our CatBoost model, the total protein, cholinesterase, apolipoprotein A1 and prothrombin activity were serological markers reflecting the synthesis and metabolism of liver. Table 1 showed that the indexes of cirrhotic ascites patients with SBP were lower than those of patients without SBP. Therefore, the function of liver synthesis and metabolism in SBP group was significantly lower than that in non SBP group. The severity of liver function damage was a predictor of SBP in cirrhotic patients with ascites. Traditional infection markers included white blood cell (WBC) count, neutrophil ratio, lymphocyte ratio, C-reactive protein (CRP) and procalcitonin. Our study showed that CRP and lymphocyte ratio had a greater contribution to the early prediction model of SBP. The SHAP force plot (Fig. 4) and previous studies^16^ all showed that CRP had a clinical value in predicting SBP infection in patients with cirrhosis ascites, but it could not be used to diagnose SBP only based on CRP level. We should put it into the prediction model to comprehensively consider its contribution to the prediction results. Lymphocytopenia performs better in predicting bacteremia in an emergency care setting than either the WBC count, neutrophil count or CRP level^17^. This was consistent with our study that lymphocyte ratio was better than neutrophil ratio in predicting SBP.

Single classification models such as logistic regression and simple decision tree model have good results in disease prediction, but there are some problems, such as weak generalization ability and poor fault tolerance. CatBoost is an ensemble learning algorithm. Different from the simple decision tree model, which use only single tree for classification, CatBoost use a series of trees, which strengthen the models ability for regression and classification. It prevents overfitting by using unbiased estimates for the gradients, so as to improve the generalization ability and robustness of the model^18^. In our study cohort, the prediction performance of CatBoost model was better than that of logistic regression and simple decision tree model (see Supplementary information). In addition, the visualization of CatBoost tree could not only make us understand the operation mechanism of the model more intuitively, but also better understand the interpretability of the model.

Machine learning model is called “black box” by many people. This means that although we can get accurate predictions from them, we cannot clearly explain or identify the logic behind these predictions. If machine learning model is to be widely used in clinic, then interpretability is particularly important. The higher the interpretability of the model, the easier it is for physicians to comprehend why a certain prediction was made and thus make an appropriate clinical decision that is in the best interest of the patient. In this study, we provided explanations for our CatBoost model using the SHAP (SHapley Additive exPlanations) method by Lundberg and Lee^19^. SHAP method is the most commonly used explainability methods. It can provide an interpretive scheme for almost all machine learning and deep learning. This method has also been used to explain the characteristics of the machine learning model in the medical field and to help understand the decision path of the model^10,11^. Although we can know the contribution of each prediction variable to the target variable from the importance matrix plot of CatBoost, it can not explain the prediction results of each observation object. However, the SHAP method can not only explain the whole data set globally, but also get explanations for individual patients, so as to understand which factors affect the individual prediction results^20^. Through the SHAP force plot (Fig. 4), we could know the contribution of each variable to the prediction results of different patients. Therefore, through the visual interpretation of machine learning model, the clinicians could understand the cause of the machine learning model's prediction, so as to use the prediction model more trust and make more beneficial clinical decisions for patients.

In this study, not only machine learning algorithm was used to establish a prediction model for the early diagnosis of first episode SBP in cirrhotic patients with ascites, but also the visualised interpretation of the machine learning black box was provided by using the SHAP values. However, there were still some limitations in this study. First, our analysis used only single-center data. The performance of the machine learning algorithm might differ for larger data sets with differently distributed patient characteristics and different institutions. As such, external validation is required to prevent overfitting. Second, in order to avoid the invasive operation caused by diagnostic abdominal puncture, we constructed the predictive model for early identification of patients with cirrhosis and ascites at risk for SBP. Therefore, only pre-paracentesis variables were analyzed in this study, and ascitic fluid characteristics, such as ascitic fluid protein level, were not assessed in the current model. Third, the algorithm learned from the input features, and some hidden relationships may have been lost because of unknown or neglected features that were not enrolled by physicians. Also, the study lacked data on the medication history of patients, such as whether the patients had used antibiotics, proton pump inhibitors, and nonselective beta-blocker. We also lacked the data to reflect the immune function of patients. Fourth, data in this study was obtained from inpatients which were at a relatively more advanced stage of the disease and were at higher risk of SBP than outpatients. Therefore, the model was only applicable to inpatients. Future prospective studies are required to evaluate the application of machine learning-based predictive models to clinical practice for achieving early diagnosis of SBP.

## Conclusions

In conclusion, we generated high-performing machine learning model that predicted the first episode SBP in cirrhotic patients with ascites using CatBoost algorithm. We also identified 6 important variables closely related to the occurrence of SBP. By the explainable machine learning methods, clinicians would be able to better understand the reasoning behind the outcome.

## Materials and methods

### Ethical statements

The study was conducted in accordance with the 1975 Declaration of Helsinki. The Ethics Committee of Mengchao Hepatobiliary Hospital of Fujian Medical University approved the study (approval no. 2020-032-01) and waived the requirement for informed consent due to the retrospective nature of the analyses.

### Study population

**Figure 5:**
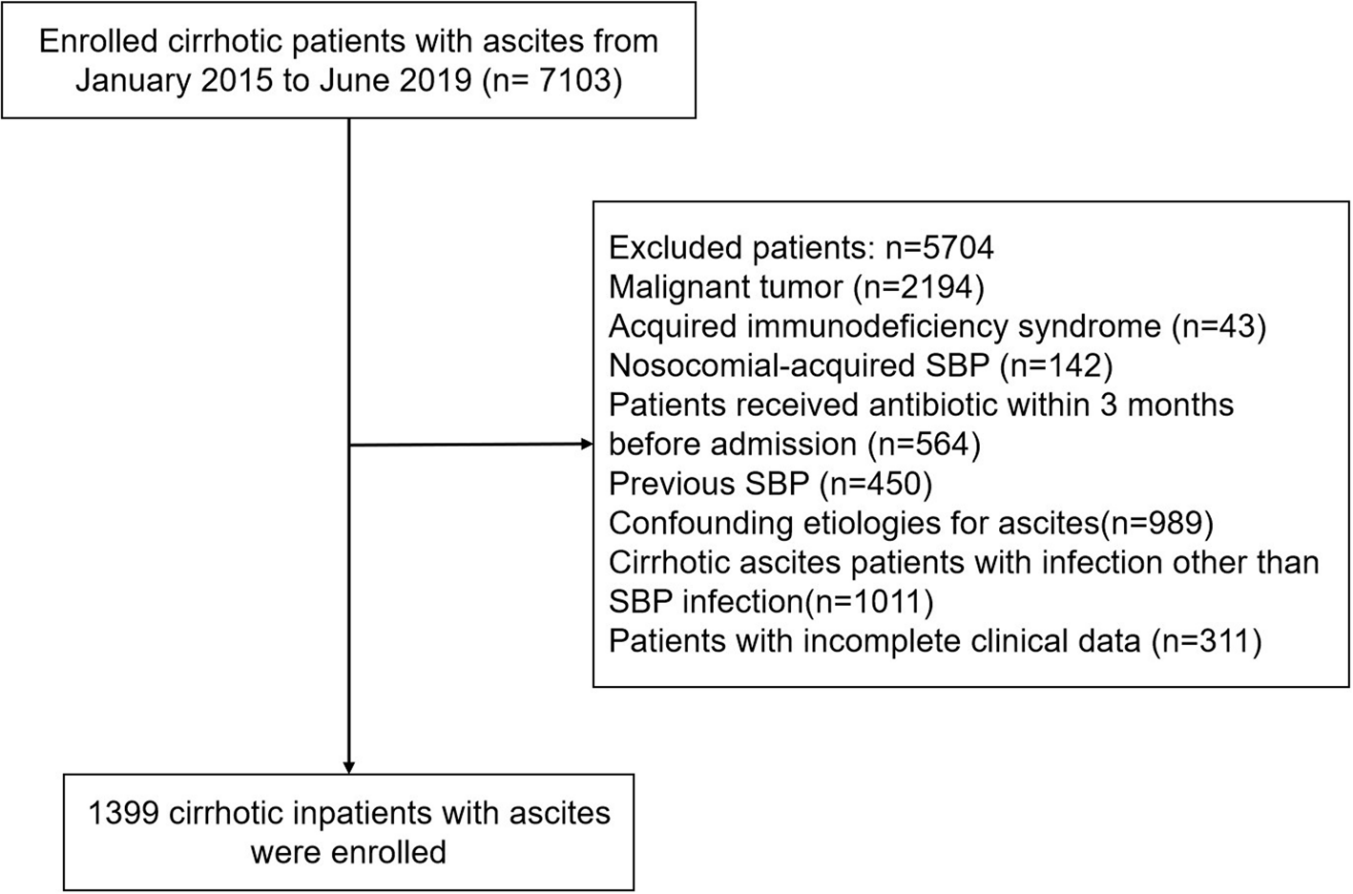
Patient screening process for model construction.

7103 adult patients (> 18 years old) with liver cirrhosis and ascites admitted for various reasons to the Mengchao Hepatobiliary Hospital (tertiary specialist hospitals) of Fujian Medical University, from January 2015 to June 2019 were included in our study. Data concerning demographic information, medical history, clinical characteristics, laboratory values, comorbidities, and physical exam findings were collected retrospectively. The exclusion criteria of model construction were: (1) malignant tumor; (2) acquired immunodeficiency syndrome; (3) nosocomial-acquired SBP; (4) patients who had antibiotic administration within 3 months before admission; (5) patients with a previous history of SBP; (6) patients with a potential confounding etiology for ascites unrelated to cirrhosis, such as peritoneal carcinomatosis, pancreatitis, tuberculosis, hemorrhage into ascites, or congestive heart failure; (7) cirrhotic ascites patients with infection other than SBP infection; (8) patients with incomplete clinical data (If a patient's data was missing more than 30 variables, we defined it as incomplete clinical data). Finally, a total of 1399 cirrhotic patients with ascites were included for the construction of the model. The patient screening process of model construction was shown in Fig. 5.

### Data collection and definitions

We retrospectively collected clinical and laboratory data on admission from available medical records and included the following variables: age, gender, weight, cirrhosis etiology, portal hypertension or not, upper gastrointestinal hemorrhage or not, hepatic encephalopathy or not, hypertension or not, diabetes or not, smoking history or not, drinking history or not, family history of liver cancer or not, total protein, white blood cell count, red blood cell count, hemoglobin, platelet count, mean platelet voulume, C-reactive protein (CRP), procalcitonin (PCT), serum creatinine, prothrombin activity(PTA) etc. Detailed information for the 46 variables are listed in Table 1.

The diagnosis of cirrhosis was based on the results of the combination of physical, laboratory, and radiologic examination results or endoscopic signs of portal hypertension. Ascites was confirmed by ultrasonography. SBP was determined according to one of the following criteria, as revised from available guidelines^13^: (1) abdominal pain and/or fever (T > 37.5 ℃), and/or abdominal tenderness and rebound tenderness (excluding secondary peritonitis); and (2) ascites polymorphonuclear cells counts ≥ 250/mm^3^ and/or positive ascites bacteria culture. A community-acquired SBP episode was considered in any case diagnosed during the first 48 h of hospitalization.

### Statistical analysis and machine learning

We used the chained equation (MICE) R package to perform the multiple imputation for dealing with the missing values. The idea of Multiple imputation is to take into account uncertainty in predicting missing values by creating multiple complete datasets. It is superior to single imputation and has been widely used in medical data analysis^21,22^. Continuous variables are presented as the means and standard deviations or medians and interquartile ranges, and categorical variables are presented as frequencies and percentages. Differences between groups were analyzed using Fisher’s exact probability test for categorical variables and Welch’s t-test (or the Wilcoxon rank-sum test) for continuous variables. Statistical analyses were performed using R version 3.6.1 (R Foundation for Statistical Computing). A *P* value < 0.05 was considered statistically significant.

For machine learning models, the CatBoost, scikit-learn and SHAP packages were used to create models and tune hyper-parameters in Python version 3.6. We used MedCalc statistical software to compare the area under the receiver operating characteristic curve (AUROC) of different models.

### Study design

The cohort of 1399 patients used for model construction was randomly divided into 2 sets—training set (70%) and validation set (30%). All models evaluation metrics were reported on the validation set, composed of the held out 448 patients who were never used in the model training. The training set was used to train a gradient boosting model (CatBoost), and the hyper-parameter was tuned using a grid search strategy with a fivefold cross-validation. The cross-validation strategy reduces overfitting of model and improves robustness.

For convenience, the CatBoost model generated with 46 variables was named MODEL-1. After a comprehensive evaluation of CatBoost importance matrix plot, SHAP summary plot and doctors' clinical experience, we selected the 6 most influential variables (explained below in Dimensionality Reduction). The CatBoost model built with these 6 variables was named MODEL-2.

For further evaluation of MODEL-1 and MODEL-2, we generated a binary classifier—whether SBP occured or not. We chose a threshold probability that maximizes F2-score of each model. Unlike the F1-score, which gives equal weights to precision (or positive predictive value (PPV)) and sensitivity (or recall), the F2-score gives more weight to sensitivity and penalizes the model more for false negatives than false positives. As our goal was to identify those patients at lower risk of SBP for reducing the use of antibiotics and invasive peritoneal puncture, our model’s metric of success should favour enhanced sensitivity. This ensured that our false negative rate was very low, that is, patients classified as low-risk were less likely to develop SBP. Sensitivity, specificity, PPV, and negative predicative values (NPV) were calculated for each binary classifier. F-score calculation is represented below and β = 2 for F2-score.

$$ F_{\beta } = \left( {1 + \beta^{2} } \right)\frac{{{\text{Precision}} \times {\text{Recall}}}}{{\beta^{2} \times {\text{Precision}} + {\text{Recall}}}} $$

We also fitted several other machine learning methods including logistic regression and simple decision tree. The CatBoost model was preferred over the other models because of its higher performance on the validation set (see Table S1; Fig. S1 of Supplementary information).

### Opening the black-box: intuitive understanding of model’s variable utility

The correct interpretation of a prediction model for machine learning is a challenge. In this study, we provide explanations for our CatBoost machine learning model using the SHAP (SHapley Additive exPlanations)^23^. The SHAP summary plot organizes the variable display from top to bottom with the most important variable at the top and least important variable at the bottom as determined by the model in question. The SHAP value greater than zero on the X axis indicates an increase in incidence of disease, while the value less than zero indicates a reduction in the incidence of disease. Each patient is represented by a dot around the horizontal variable line. Each dot’s color reflects the value of the patient’s variable which is scaled to a normal color coded distribution (red is larger and blue is smaller).

The SHAP summary plot is a global explanation of the prediction results of the data set, and the SHAP force plot explains the prediction results of each individual patient to us. The SHAP force plot can be used to visualize the Shapley value for each feature as a force, which either increases (positive value) or decreases (negative value) the prediction from its baseline^19^. The baseline for the Shapley value is the average of all predictions, which, in our case, is the average incidence rate of the validation set predicted by the model we build. In order for us to understand why the machine learning model came to a certain conclusion, we will use the SHAP explanation force plot to explain individual prediction results of 4 randomly selected patients from the validation set.

### Dimensionality reduction

Dimensionality reduction is an important process in machine learning model development. In CatBoost importance matrix plot and SHAP summary plot, we first selected the top 10 most influential variables according to the variable influence ranking. Then, according to the feature importance measures, the variable with the lowest importance measures was eliminated each time to build the model. In the validation set, the AUROC values of each model were compared to obtain the simplest and optimal model.

### Model generalization ability evaluation

In order to proved that our models can predict the presence of SBP in patients with cirrhotic ascites complicated with other infections. We evaluated the predictive performance of the models in 1011 patients with cirrhotic ascites with infection other than SBP.

## Supplementary Information

Supplementary Information.

## Abbreviations

SBP: Spontaneous bacterial peritonitis
AUROC: Areas under the receiver operating characteristic curve
SHAP: Shapley additive explanation
HBV: Hepatitis B virus
CRP: C-reactive protein
PTA: Prothrombin activity
