# Supplementary material for: Explainable machine learning model for predicting spontaneous bacterial peritonitis in cirrhotic patients with ascites
Source: Sci Rep. 2021 Nov 4;11:21639. doi: 10.1038/s41598-021-00218-5 (PMC8569162; doi:10.1038/s41598-021-00218-5)

**Supplementary Material**

Table 1: Performance of Machine Learning Models in predicting first episode SBP of patients with cirrhotic ascites in the validation set.

| **Models** | **AUROC** | **95% Confidence Interval** | **p-value** |
| --- | --- | --- | --- |
| CatBoost model | 0.822 | 0.784 ~ 0.856 |  |
| Logistic regression model | 0.792 | 0.751 ~ 0.829 | 0.011 |
| Decision tree model | 0.774 | 0.733 ~ 0.812 | 0.001 |

Figure 1: Receiver Operator Characteristic (ROC) curves for Machine Learning Model (Validation Set).


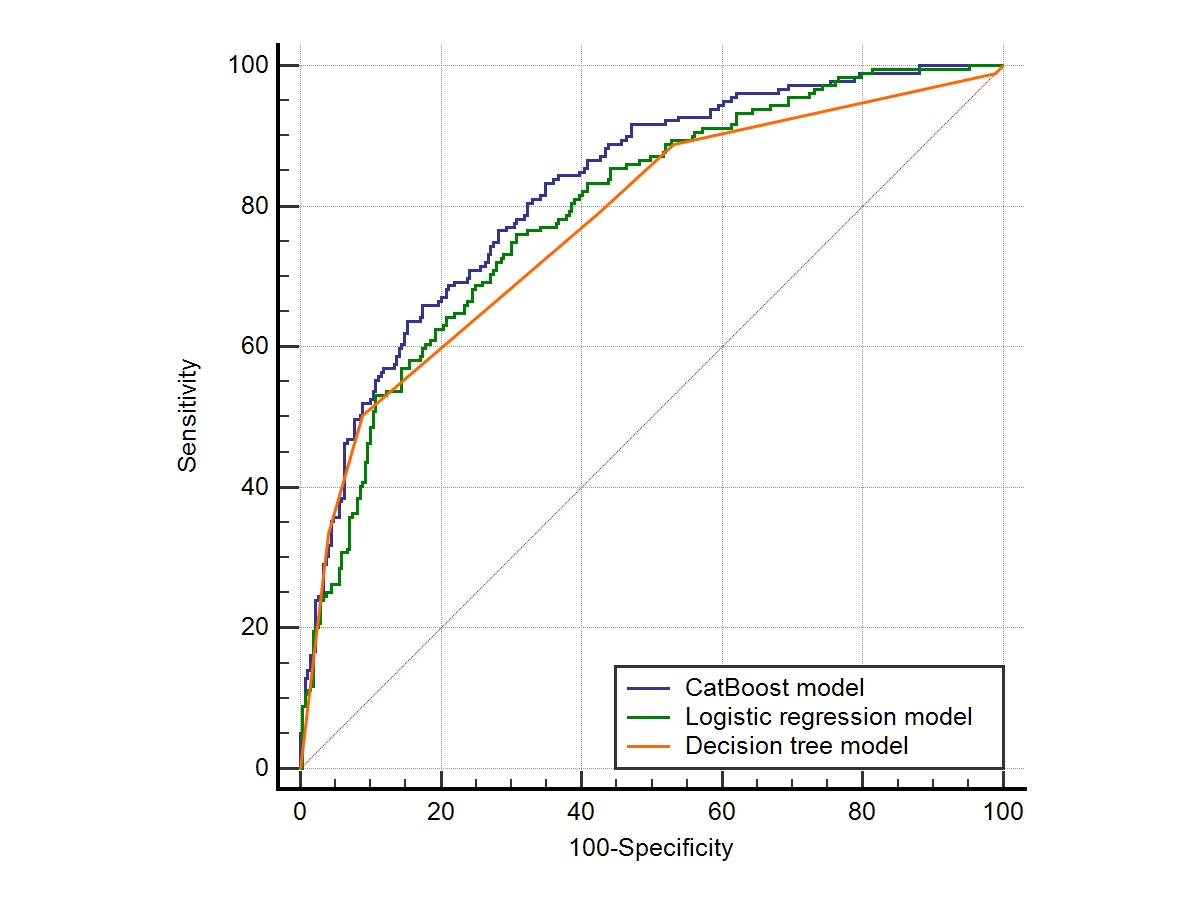


Table 2: Comparison of AUROC of different models in the process of feature selection

| **Number of variables** | **Variables used to build the model** | **AUROC** | **95% Confidence Interval** |
| --- | --- | --- | --- |
| 11 | Protein, CRP, PTA, Cholinesterase, Apoa1, LYR, Sodium, TBA, WBC, HDL, ACLF | 0.822 | 0.783 to 0.856 |
| 10 | Protein, CRP, PTA, Cholinesterase, Apoa1, LYR, Sodium, TBA, WBC, HDL | 0.823 | 0.784 to 0.857 |
| 9 | Protein, CRP, PTA,Cholinesterase, Apoa1, LYR, Sodium, TBA, WBC | 0.822 | 0.783 to 0.856 |
| 8 | Protein, CRP, PTA, Cholinesterase, Apoa1, LYR, Sodium, TBA | 0.821 | 0.782 to 0.855 |
| 7 | Protein, CRP, PTA, Cholinesterase, Apoa1, LYR, Sodium | 0.820 | 0.781 to 0.855 |
| 6 | Protein, CRP, PTA, Cholinesterase, Apoa1, LYR | 0.822 | 0.783 to 0.856 |
| 5 | Protein, CRP, PTA, Cholinesterase, LYR | 0.817 | 0.778 to 0.852 |
| 4 | Protein, CRP, PTA, Cholinesterase | 0.814 | 0.774 to 0.849 |
| 3 | Protein, CRP, PTA | 0.801 | 0.761 to 0.837 |

Protein: Total protein; CRP: C-reactive protein; PTA: Prothrombin activity; Apoa1: Apolipoprotein A1; LYR: Lymphocyte ratio; TBA: total bile acid; HDL: High density lipoprotein; WBC: white blood cell count; ACLF: acute(sub-acute)-on-chronic liver failure.

Figure 2: Visualization of the top five CatBoost trees in MODEL-2


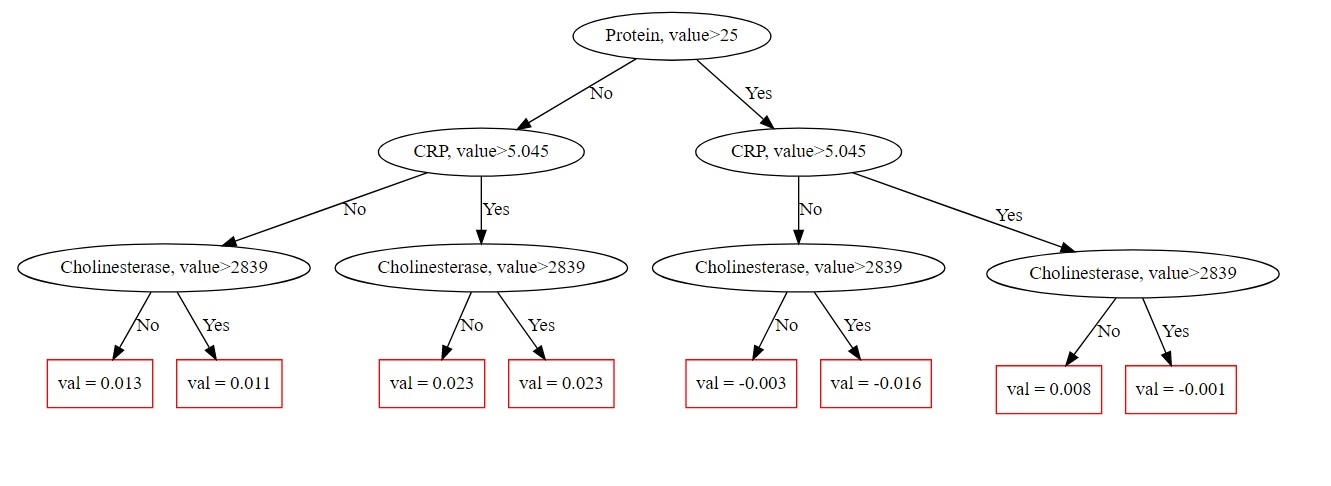


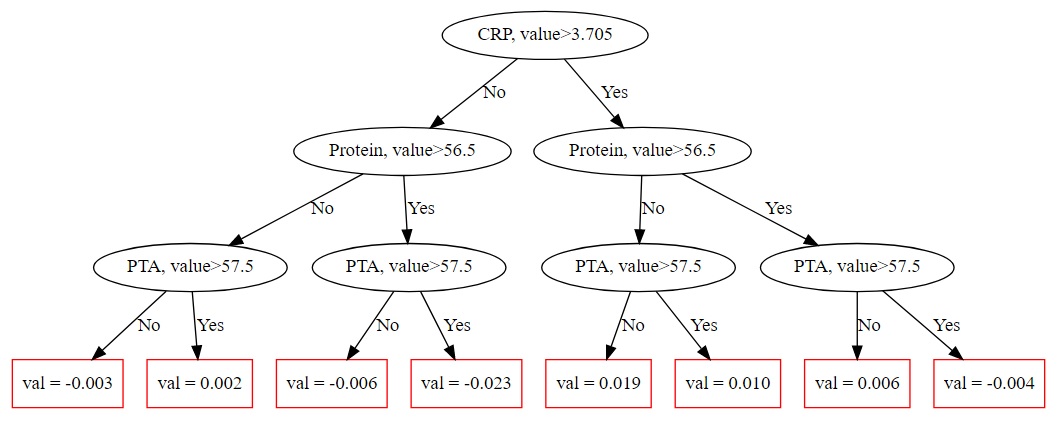


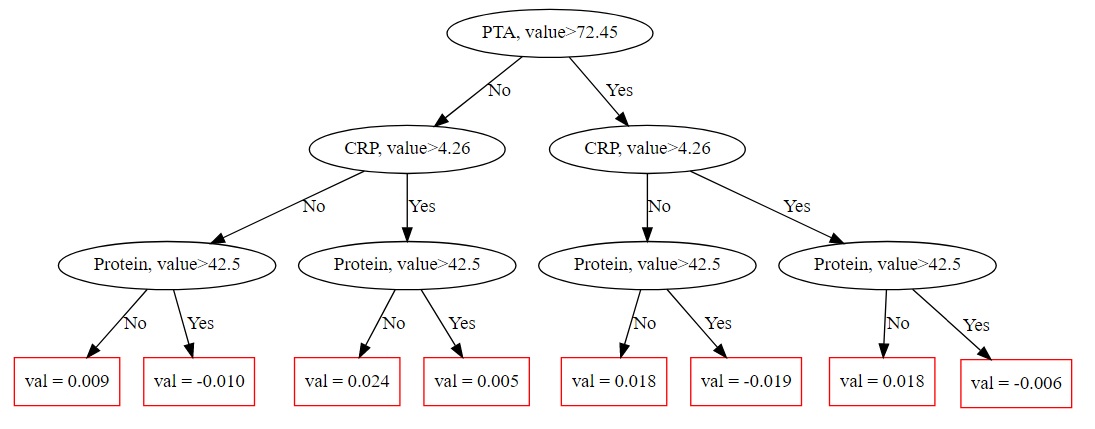


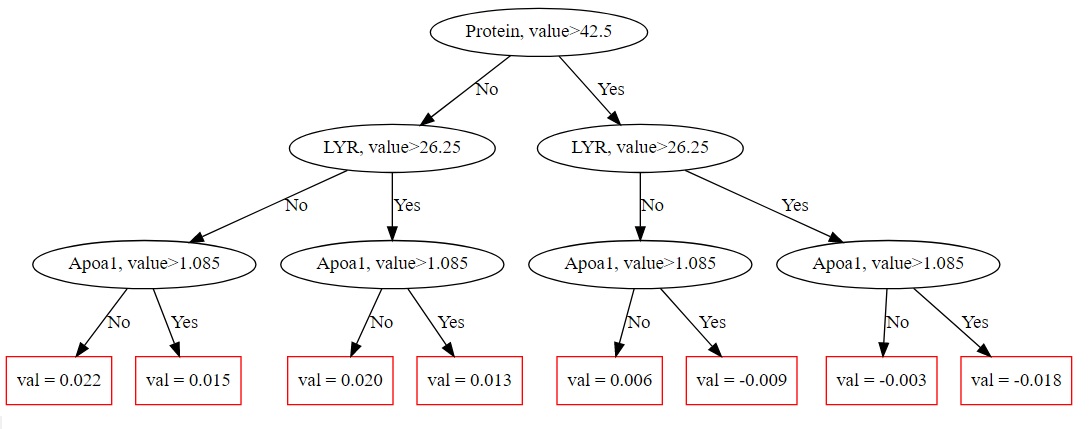


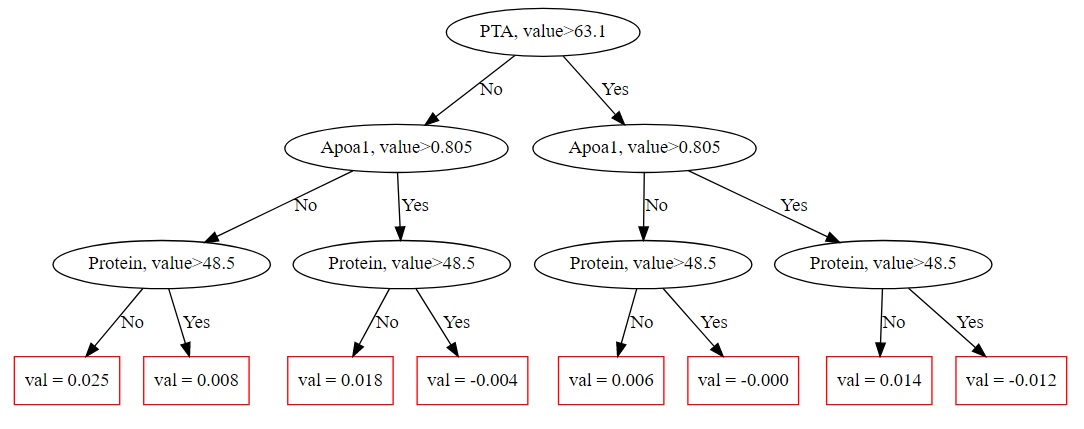

Supplement: Supplementary file 1 — Supplementary Information. [file 41598_2021_218_MOESM1_ESM.doc]
